# Promoting Evidence-Based Decision Making in a Local Health Department, Pueblo City–County, Colorado

**DOI:** 10.5888/pcd12.140507

**Published:** 2015-06-25

**Authors:** Anna K. Hardy, Christine Nevin-Woods, Sylvia Proud, Ross C. Brownson

**Affiliations:** Author Affiliations: Christine Nevin-Woods, Sylvia Proud, Pueblo City–County Health Department, Pueblo, Colorado; Ross C. Brownson, Prevention Research Center in St. Louis, Brown School, Washington University in St. Louis, Division of Public Health Sciences and Alvin J. Siteman Cancer Center, Washington University in St. Louis School of Medicine, St. Louis, Missouri.

## Abstract

**Background:**

Evidence-based decision making (EBDM) is an effective strategy for addressing population health needs. Assessing and reducing barriers to using EBDM in local health departments may improve practice and provide insight into disseminating EBDM principles among public health practitioners.

**Community Context:**

Administrative leaders at the Pueblo City–County Health Department, Pueblo, Colorado, used a systematic approach for implementing EBDM. Research partners engaged staff to understand factors that increase or deter its use.

**Methods:**

A survey was distributed to staff members at baseline to identify gaps in administrative and individual practice of EBDM. In-depth interviews were also conducted with 11 randomly selected staff members. Results were shared with staff and administration, after which activities were implemented to improve application of EBDM. A follow up survey was administered 1 year after the initial assessment.

**Outcome:**

Survey data showed evidence of progress in engaging and educating staff members, and data showed improved attitudes toward EBDM (ie, several items showed significant improvement from baseline to follow-up). For example, staff members reported having the necessary skills to develop evidence-based interventions (73.9%), the ability to effectively communicate information on evidence-based strategies to policy makers (63.0%), access to current information on improving EBDM processes (65.2%), and a belief that evidence-based interventions are designed to be self-sustaining (43.5%).

**Interpretation:**

Within a local health department in which leaders have made EBDM a priority, addressing the culture and climate of the department may build EBDM. Future research may provide insight into tailoring EBDM within and across local health departments.

## Background

Evidence-based decision making (EBDM) provides a framework to address many critical challenges (eg, setting priorities, making efficient use of resources) facing the public health system (1). EBDM is a process used to determine the best intervention for a population; it is rooted in community needs, practitioner experience, and existing evidence (2). EBDM produces high-quality information on what works in populations, resulting in implementation of successful programs and policies, greater workforce productivity, and more efficient use of funding (1). Public Health Accreditation Board Standard 10 requires the use and dissemination of evidence (3), which contributes to the momentum for EBDM.

Although the key tenets of EBDM are now well established (1,4,5), information is sparse on how to implement EBDM concepts in day-to-day public health practice. Research shows that EBDM practice does not happen organically (5); there are common barriers to engaging local health departments in EBDM. These include philosophical differences between practitioners and researchers; lack of time, money, or incentives; and insufficient organizational support (5). One survey of 447 state and territorial health care practitioners treating chronic disease showed that the strongest barriers to using EBDM were organizational factors (6). Other national data show the importance of agency size and make-up (ie, types of staff members and their qualifications) in EBDM (7,8).

Organizational structures and activities associated with EBDM performance, called administrative evidence-based practices (AEBPs) (5), fall into 5 domains: workforce development, leadership, organizational culture and climate, relationships and partners, and financial characteristics of the agency (7). This article describes an assessment of AEBPs and individual staff-member factors in a local health department involved in adopting EBDM, the activities related to promoting EBDM that took place over 1 year, and the impact of those activities on staff attitudes and perceptions toward EBDM.

## Community Context

The Pueblo City–County Health Department (PCCHD), located in southeastern Colorado, was selected for this case study because of the public health director’s interest in establishing EBDM as standard practice throughout the department. Initial contact was made between the director and the research team through an EBDM training. PCCHD is a mid-sized health department governed by a local board of health. It serves a population of 159,000 that is 54% non-Hispanic white and 42% Hispanic. Approximately 18% of residents in PCCHD’s jurisdiction have incomes below the federal poverty limit, and 72% have completed high school (9). PCCHD has approximately 90 employees in 8 departments: Women, Infants, and Children program; budget and finance; vital statistics; laboratory; environmental health; community health services; administration and disease prevention; and emergency preparedness. The department’s operating budget is $6.7 million, with funding from local, state, and federal government and from private foundations. At the start of this study, the director was a physician with a master’s degree in public health who had been at PCCHD for 22 years. The director had invested in building the EBDM capacity of her staff and wished to create the infrastructure and organizational culture that would make EBDM the norm in the department. The objective of this study was to improve EBDM among PCCHD staff members, which would be evidenced by changes in staff members’ perceptions and knowledge of EBDM from baseline to follow-up 1 year after community engagement began.

## Methods

This study used a mixed-methods approach consisting of a quantitative survey, individual staff member interviews, and a focus group. The survey was based in part on the project team’s prior research (5,10). We measured a set of AEBPs and EBDM skills by asking respondents to rank them according to importance and availability.

On the basis of results of the initial quantitative survey, gaps between scores for importance and availability and low-ranking AEBPs were identified, and qualitative interview questions were developed to explore those gaps. The individual interviews consisted of 24 questions, which were reviewed for comprehension with 1 staff member and 1 public health professional from a different local health department.

In addition to the individual interviews, 1 focus group was conducted and opened to all staff members in order to allow those who were not interviewed a chance to express their views. A shortened 12-question version of the individual interview was given to focus group participants. The institutional review board of Washington University in St. Louis approved this project.

### Data collection

In February 2013 a baseline survey was sent to 74 PCCHD staff members chosen by administrators on the basis of how pertinent EBDM was to the type of work the staff members performed. Email addresses were acquired from PCCHD, and surveys were distributed via individualized links generated using the Qualtrics online research suite (www.qualtrics.com). PCCHD administrators encouraged the staff to complete the surveys through face-to-face interaction or email. No incentives were given. The follow-up survey contained the same questions as the initial one with an additional 4 questions to assess the impact of activities that occurred during the time between surveys. The follow-up survey was administered to all staff members who received the initial survey and were still employed by PCCHD in April 2014 (n = 46).

Drawing from respondents to the quantitative survey, a random sample of 11 staff members was selected to participate in a semistructured face-to-face interview.

### Data analysis

We calculated frequencies and proportions for both participant characteristics and for the various survey items. Several categorical items were recoded as binary (1 = yes, 0 = no or do not know), and χ^2^ tests were used to assess differences in proportions between the 2 surveys. To examine mean differences, we conducted paired sample *t* tests (repeated measure) for items related to organizational and individual skills. To describe the magnitude and direction of any significant differences, we calculated effect sizes. Cohen’s *d* values were calculated for mean differences observed in *t* tests. Cohen suggests the following effect ranges for *d*: 0.2, small; 0.5, medium; and 0.8, large (11). In addition, we calculated Cramér’s *V* values for effect sizes for differences in proportions. Cramér’s *V* may be interpreted as 0.1, small effect size; 0.3, medium effect size; and 0.5, large effect size (12). All analyses were conducted in SPSS version 20 (IBM Corp) or Microsoft Excel version 2010 (Microsoft Corp).

All responses were recorded and were confidential and anonymous. The interviewer transcribed the qualitative interviews. Open coding was completed for each transcript to ascertain general themes. The interviewer next used focused charting to develop a codebook that was distributed to a team of researchers. The team made changes to more adequately capture codes and subcodes. The final codebook was then used by the interviewer to analyze each script. In addition, 1 research assistant analyzed 3 scripts for interrater reliability. Discrepancies between reviewers were minimal. A nonparticipant transcribed the content of the focus group, which was then analyzed by verbally verifying content for accuracy among the group, and then capturing major themes and concluding remarks by using open coding.

## Outcome

### Initial survey, staff interviews, focus group

Of the 74 staff members who received the baseline survey, 59 completed it, an 80% response rate. The majority of respondents were technical experts (ie, planner, grant writer, epidemiologist, health educator, environmental health specialist, emergency preparedness educator [49.2%]) or other (23.7%), which included nurses, a registered dietician, administrative assistants, accountants, and the vital statistics staff. On average, participants had spent 5.7 years in their current position (standard deviation [SD], 6.9), and 9.5 years in public health (SD, 8.5). Two AEBP domains ranked lowest in the surveys, leadership and organizational climate and culture (Table 1). The personal skill for which respondents reported the highest proficiency was the ability to develop evidence-based interventions. Staff members reported that training in EBDM would most encourage them to use EBDM (Table 2).

**Table 1:** Responses of Administrative and Individual Staff Members to Baseline (N = 74) and Follow-Up (N = 46) Surveys, Evidence-Based Decision Making Practices (EBDM), Pueblo City–County, Colorado, Health Department, February 2013 and April 2014

EBDM Effect on Work Practice, by Domain	Baseline Survey, February 2013	Follow-Up Survey, April 2014	*P* Value^a^	Cramér’s *V*	Cohen’s *d*
**Administrative practice**					
**Leadership, mean (SD)**					
Enhanced my ability to lead in EBDM^b^	5.6 (1.2)	6.0 (1.1)	.14	—	0.3
Encouraged use of EBDM^b^	5.6 (1.5)	5.9 (1.3)	.27	—	0.2
Fostered staff participation in decision making^b^	5.0 (1.6)	4.9 (1.9)	.77	—	0.1
Hires people with public health degree^b^	4.3 (1.4)	4.7 (1.2)	.14	—	0.3
Hires people with experience in public health^b^	4.6 (1.3)	4.8 (1.2)	.53	—	0.1
Overall domain^c^> (%)	41.0	51.7	—	—	—
**Organizational climate and culture of agency, mean (SD)**					
Culture that supports EBDM^b^	5.3 (1.5)	5.5 (1.4)	.48	—	0.1
Access to current research evidence^b^	5.3 (1.4)	5.8 (1.2)	.11	—	0.3
Promotes life-long learning^b^	5.7 (1.5)	5.7 (1.6)	.98	—	0.0
Access to EBDM information relevant to community needs^b^	5.4 (1.3)	5.7 (1.6)	.36	—	0.2
Overall domain^b^, %	51.2	70.7	—	—	—
**Financial characteristics of agency, N (%)**					
Funded through several sources^d^	41 (95.3)	43 (95.6)	.96	0	—
Allocated resources for quality improvement^d^	9 (20.0)	30 (65.2)	<.001	0.5	—
Overall domain^c^, %	55.9	79.4	—	—	—
**Workforce development, N (%)**					
Access to training in EBDM^d^	23 (57.5)	38 (92.7)	<.001	0.4	—
Access to training in quality improvement processes^d^	19 (42.2)	32 (71.1)	.01	0.3	—
Access to training in management practices^d^	26 (57.8)	24 (52.2)	.59	0.1	—
Access to training in performance assessment^d^	29 (64.4)	28 (60.9)	.73	0	—
Access to current information on improving EBDM processes^b^, mean (SD)	4.8 (1.4)	5.5 (1.4)	.03	—	0.5
Overall domain^c^, %	59.2	66.1	—	—	—
**Relationships and partnerships, mean (SD)**					
Partnerships have missions that align with agency^b^	5.5 (1.2)	5.5 (0.9)	.96	—	0.0
Important to have partners who share resources^b^	5.4 (1.1)	5.8 (1.1)	.12	—	0.3
Important to develop partnerships with both health and other sectors^b^	6.2 (0.9)	6.4 (0.7)	.26	—	0.2
Overall domain^c^, %	59.4	73.2	—	—	—
**Individual practice, mean (SD)**					
I have skills necessary for developing evidence-based interventions^b^	5.3 (1.3)	5.9 (0.8)	.02	—	0.4
I can effectively communicate information on evidence-based strategies to policy makers^b^	4.9 (1.5)	5.6 (1.1)	.02	—	0.4
I feel I need to be an expert on many issues in order to effectively make evidence-based decisions^b^	4.7 (1.5)	4.2 (1.3)	.09	—	0.3
My fears about job security prevent me from using EBDM^b^	2.5 (1.6)	2.3 (1.4)	.51	—	0.1
I feel evidence-based interventions are packaged in a way I can use them^b^	4.7 (1.2)	5.0 (0.8)	.21	—	0.2
I feel evidence-based interventions are designed in a way to be self-sustaining^b^	4.7 (1.0)	5.2 (1.1)	.04	—	0.5

Abbreviation: SD, standard deviation; —, not applicable.

a *P* values are reported for χ^2^ test, paired-sample *t* test, or independent *t* tests.

b Seven-point Likert scale response option (7 = strongly agree; 1 = strongly disagree).

c Expressed as a percentage of yes for yes/no/don’t know and “strongly agree” or “agree” for Likert response options within each domain.

d Yes/no/don’t know response option.

**Table 2:** Rankings of Activities That Would Most Encourage Staff to Use Evidence-Based Decision Making Practices (EBDM),^a^ Baseline and Follow-Up Surveys of Administrative and Individual Staff Members (N = 46), Pueblo City–County, Colorado, Health Department, February 2013 and April 2014

Rank^a^	Baseline Survey, February 2013	Follow-Up Survey, April 2014
1	Training on EBDM	Positive feedback or encouragement to use EBDM
2	Placing high priority on EBDM by leaders in my agency	Placing high priority on EBDM by leaders in my agency
3	Positive feedback or encouragement to use EBDM	Training on EBDM
4	Professional recognition for use of EBDM	Professional recognition for use of EBDM
5	Performance evaluation that considers use of EBDM	Performance evaluation that considers use of EBDM

a The 5 items were ranked on a scale of 1 to 5 (1 = highest ranking; 5 = lowest ranking).

Among 11 staff members interviewed, the length of employment at PCCHD ranged from 1.5 to 21 years. Nine interviewees were line staff, and 2 held manager or supervisory roles. Five of 8 departments were represented.

Qualitative findings were grouped into benefits and barriers to EBDM use and action steps to promote EBDM at PCCHD (Table 3). The benefits to using EBDM cited most often were that it fosters a targeted use of limited resources and that it improves service to the community. Barriers to EBDM use at PCCHD were a lack of capacity and internal and external inflexibility (ie, resistance to change).

**Table 3:** Responses of Administrative and Individual Staff Members (N = 46) to Open-Ended Interviews, Evidence-Based Decision (EBDM) Making Practices, Pueblo City–County, Colorado, Health Department, June–July 2013

Theme	Response
Benefits of using EBDM	Better use of resources: “[Using EBDM] we would stop doing things that aren’t essential and that would free up resources to do something else or do it right . . . you look at the numbers and do what’s right.”
	Better service to community: “ . . . letting people know the health department is doing this because we . . . are a credible agency and we want to be taken seriously, we value the information that we’re giving you and we want to make sure that what we are doing is going to work for you.”
Barriers to using EBDM	Lack of capacity (finances, personnel, time): “There’s not enough capacity in public health to do everything that evidence would show us would be a wise approach.”; “It’s really hard; they (funding agencies) are telling us, ‘use evidence-based, but we’re not going to give you . . . in fact we’re going to give you less money.’”; “[W]e’ve been short staffed, so it’s been difficult.” “Often times that gets difficult when you’re down at the level of doing a lot of work because you’re trying to get through the day and deal with all the things that come up.”
	Internal inflexibility: “Being that I guess we’ve never done [EBDM] in the other programs, it might be a little difficult to get some people on board with that because . . . people have been doing things a certain way for years.”
	External inflexibility: “We’ve always done things this way in Pueblo.”
How to promote EBDM to staff	Explain how EBDM fits within each job description/department: “I think every program is so different that you can’t really just throw one instance of . . . the proof of EBDM for this program. I think each program would have to have its own SOP [standard operating procedure] written up. Maybe the directors would be able to explain to the people that work in that department . . . the process to go through.”
	EBDM is a process, not program: “I think the one thing that happened . . . that I found very frustrating, and I think other people did too, is [EBDM] became like a buzz word; . . . it had to be a program, not just evidence-informed, that it had to be like this evidence-based program in order to be successful. And I think that turned people off.”
	Model use of EBDM: “[Hearing administration’s] use of evidence-based programming and their successes with it would help promote an environment that supports EBDM.”
	Listen to and follow-up on staff suggestions: “So actually following through on those ideas and saying ‘Well, we could really fix this’ in a meeting, but then everyone going back to their silo and never talk[ing about it] is the death of all that [feedback].”
	Positive reinforcement: “I mean we wouldn’t get into public health for the money or anything like that. So I think just hearing a ‘Good job!’”
Training	Hands-on experience that pertains to their individual interests: “So I think the more we can get away from things like Webinars [the better] . . . So those types of [training] of hands-on with practical application are very good.”

Staff recommended several action steps for improving the use of EBDM at PCCHD. First, health department leaders and managers should clearly communicate EBDM messages to various departments and model EBDM as a process, in a manner that crosses categorical programs. A second suggestion was to listen to and follow up on staff suggestions. Third, staff members requested positive feedback from leadership to encourage use of EBDM. Finally, staff members wanted to understand the relevance of EBDM to their work. As for training in EBDM, and consistent with adult education theories (13), staff respondents indicated that the most useful training experiences are those that are hands-on and have practical application to their professional duties.

Focus group findings were also clustered into 3 general topics related to EBDM: benefits, barriers, and action steps. Respondents said that benefits to using EBDM in a local health department were “smart business”: EBDM can improve programs, help government agencies stay accountable, determine program effectiveness, and improve efficiency. The barriers were internal and external politics, funding and time limitations, personal agendas, differences in recognizing the value of EBDM across departments within local health departments, and limited knowledge and self-efficacy for using the process. When asked, “If you were the administrator and wanted to make EBDM a priority, what would you do to bridge the gap between benefits and barriers to EBDM?,” the overwhelming majority agreed that securing sustainable funding, rather than grant-focused funding, would be the most beneficial. Other responses included explaining how EBDM is pertinent to each department, demonstrating and explaining specific steps in EBDM, and improving the public’s understanding of the purpose of public health.

### Activities between surveys

Baseline survey findings were presented to key administrators at PCCHD, the local board of health, and to all PCCHD staff in several meetings. Presentations were a collaborative effort between the research team and PCCHD administration and staff. The results were shared in order to engage the PCCHD staff in developing policies and activities aimed at reducing the barriers to EBDM.

The following is a summary of the recommendations given at the conclusion of the research assistant’s (A.H.’s) time with PCCHD:

- Clearly define and differentiate workforce improvement initiatives.
- Pay attention to EBDM messaging, modeling, and follow-up activities, because they are vital for the success of EBDM.
- Ensure every person and division understands the value of EBDM and how each staff member can use the process to improve service.
- Ensure that administration and management regularly discusses EBDM ideas and progress in meetings.
- Create an EBDM promotion team from among staff members trained in EBDM.

Two-thirds of the staff agreed or strongly agreed that these recommendations accurately captured staff behaviors and feelings about EBDM.

Following the assessment period, the research team maintained communication with the PCCHD community via conference calls and email to learn about, and track progress on, their plans for addressing improvement in the uptake of EBDM. Notably, many strategies were operationalized through collaboration between staff members and external partners. For example, PCCHD partnered with a state university to tailor a 3-day EBDM training for PCCHD staff that was based on an established training model (2). Agency leaders invited all staff members who held positions that allowed them to make evidence-based decisions. An EBDM team in PCCHD made up of staff members with EBDM training and experience was identified as a resource for staff members to contact for consultation as they learned the EBDM process. Administrators clarified messages about EBDM (ie, that it is a process, not a specific program) as the results of the survey were shared with staff members. To minimize confusion, messages and concepts related to EBDM, quality improvement, and the plan-do-check-act model (a management method used for continuous improvement) were merged into 1 decision flowchart and presented to the entire staff (Figure). EBDM was then incorporated into the 5-year strategic plan by the PCCHD strategic planning committee, where it now states that PCCHD “will implement a quality improvement plan using EBDM.” The local board of health was asked to promote the process by asking the PCCHD administration to formalize the use of EBDM in PCCHD as they made decisions on policies and programs. For example, EBDM language was incorporated into several PCCHD policies, namely the formal grant process, personnel policies, the strategic plan, and supervisor guidelines (in process).

**Figure:**
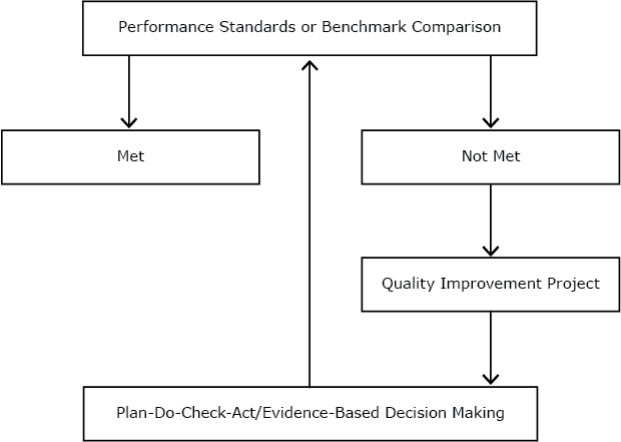
Integration of evidence-based decision making, quality improvement, and plan-do-check-act at Pueblo City–County Health Department, Colorado.

### Follow-up survey

The follow-up survey was sent to the 46 staff members who were respondents to the initial survey and who were still employed at PCCHD; the survey had a 100% response rate. Like the initial survey, the majority of respondents were technical experts (50.0%) or other (23.9%). Participants had an average of 6.9 years in their current position (SD, 7.0), and 10.3 years in public health (SD, 8.5). Between surveys, 55.9% of the respondents participated in an all-staff meeting where information on EBDM was presented, and 52.5% attended the tailored 2-day training.

Significant changes were made in the AEBPs in the follow-up survey for access to training in EBDM and quality improvement processes, access to current information on EBDM, and knowing PCCHD allocated resources for quality improvement (Table 1). Significant improvement in individual skills assessed in the follow-up survey were having skills in developing evidence-based interventions, possessing communication skills, and acknowledging the self-sustaining nature of evidence-based interventions.

Changes between pre- and post-survey data on activities that would most encourage staff to use EBDM (Table 2) are noteworthy. For example, at baseline survey, training in EBDM was ranked as the activity most likely to motivate staff members to use EBDM. At the post-training survey, training in EBDM had shifted to third highest priority, and encouragement in use of EBDM was ranked highest. This change suggests that, after administrators provided training opportunities, the next phase in increasing use of EBDM was for administrators to provide positive feedback or encouragement in employees’ use of EBDM.

The success of PCCHD staff engagement was largely due to administrative support. Administrators were dedicated to delivering messages about EBDM in ways their staff members found helpful. They combined EBDM and quality improvement processes in order to make using both less confusing. The partnership between a state university and the PCCHD was crucial to delivering the 2-day EBDM training, which was tailored to address the needs identified by staff during the assessment phase.

## Interpretation

This case study provides evidence that a local health department can improve attitudes toward EBDM among its staff in a relatively short time. First, through qualitative and quantitative evaluation, we were able to understand the barriers a local health department faced in using EBDM. Second, we showed evidence of staff engagement in improving attitudes and perceptions of EBDM principles as shown by two-thirds of the staff reporting that the team accurately captured their attitudes toward EBDM and significant improvement in several quantitative outcomes.

This study had several limitations. The main limitation relates to community engagement in that not all staff members were included in the survey. Because the pool of respondents was chosen by PCCHD administrators on the basis of job descriptions, results may be skewed in favor of EBDM practice. Another limitation is that 17% (10/59) of the original sample was lost to follow-up because they were no longer employed at PCCHD. In addition, organizational activities occurred during the year from baseline to follow-up, and activities occurring close in time to follow-up data collection may have been recalled more clearly, allowing the potential for bias. Finally, the context-specific nature of a case study limits the generalizability of any findings to other local health departments.

The activities conducted by PCCHD show evidence of improvement in attitudes and perceptions toward EBDM over 1 year. Previous studies suggest that strong leadership is a key ingredient in creating a work climate conducive to EBDM in a local health department (14). At baseline, the PCCHD staff members ranked organizational culture and climate as one of the lowest AEBPs. However, in 1 year, through continuing community engagement, and with an administration committed to making EBDM standard practice, organizational culture and climate were no longer among the lowest ranked AEBPs. The workforce development efforts described here may have influenced this improvement. Enhancing the climate and culture of a local health department is likely a pivotal activity for enhancing the uptake of EBDM (6,7).

This case study of a local health department community builds on previous work (14,15) by providing insight into steps that may improve EBDM adoption within local health departments. Additional observational studies may examine the dissemination of EBDM among PCCHD’s network with other local health departments to assess dissemination and implementation within the public health system (16). EBDM training might focus on hands-on case studies and use of a range of potential resources (4) that pertain to various topics covered by different departments within local health departments and other community-level agencies (eg voluntary health organizations). In practice, leadership may seek to concentrate on fostering a culture and climate conducive to using EBDM by establishing its relevance to all staff members, reiterating that EBDM includes community and practitioner input, and including staff members in key decision making processes.
